# Redefining the Septal L-Strut to Prevent Collapse

**DOI:** 10.1371/journal.pone.0153056

**Published:** 2016-04-13

**Authors:** Jung-Seob Lee, Dong Chang Lee, Dong-Heon Ha, Sung Won Kim, Dong-Woo Cho

**Affiliations:** 1 Department of Mechanical Engineering, POSTECH, Pohang, Korea; 2 Department of Otolaryngology–Head and Neck Surgery, The Catholic University of Korea, College of Medicine, Seoul, Korea; Navoadaya Dental College and Hospital, mantralayam Road, INDIA

## Abstract

During septorhinoplasty, septal cartilage is frequently resected for various purposes but the L-strut is preserved. Numerous materials are inserted into the nasal dorsum during dorsal augmenation rhinoplasty without considering nasal structural safety. This study used a finite element method (FEM) to redefine the septal L-strut, to prevent collapse as pressure moved from the rhinion to the supratip breakpoint on the nasal dorsum and as the contact percentage between the caudal L-strut and the maxillary crest changed. We designed a 1-cm-wide L-strut model based on computed tomography data. At least 45% of the width of the L-strut in the inferior portion of the caudal strut must be preserved during septoplasty to stabilize the septum. In augmentation rhinoplasty, the caudal L-strut must either be preserved perfectly or reinforced to prevent collapse or distortion of the L-strut. The dorsal augmentation material must be fixed in an augmentation pocket to prevent movement of graft material toward the supratip breakpoint, which can disrupt the L-strut. We conducted a numerical analysis using a FEM to predict tissue/organ behavior and to help clinicians understand the reasons for target tissue/organ collapse and deformation.

## Introduction

The prevalence of nasal surgery as a cosmetic treatment (including septorhinoplasty) is rising. Septal cartilage is frequently resected to correct a deviated nasal septum, to straighten the nasal dorsum, and to harvest augmentary material for septorhinoplasty. Preserving the L-shaped septal cartilage, known as the cartilaginous L-strut, has long been considered an important element during nasal surgery[1,2]. The septal L-strut is classically defined as a segment of the dorsal and caudal septal cartilage of at least 1 cm in width[3,4]. Moreover, dorsal augmentation to correct a low-profile nose using silicone, Gore-tex, septal cartilage, costal cartilage, or auricular cartilage is the most commonly performed nasal procedure in Asian populations. Numerous materials and methods are used for augmentation rhinoplasty and septoplasty[5–8]. However, evidence regarding septal safety after septal resection or dorsal augmentation rhinoplasty[1,2,9,10] is limited to the clinical outcomes of case series and individual physician’s opinions. Therefore, it is important to examine the reasons for deformation or collapse of the L-strut.

The finite element method (FEM) has become more available with advances in computer technology. The field of FEM has increased to include analyses of laryngeal voice, middle-ear mechanics, and nasal mechanical structure[10–18]. However, few studies have used FEM technology to examine the nasal septum.

We previously presented preliminary data regarding the safety of the septal L-strut when resecting the caudal segment of the L-strut to correct a caudal septal deviation[19,20]. The aim of the present study was to elucidate the detailed distribution of stress in the L-strut after the inferior portion of the L-strut caudal component, which is connected to the maxillary crest, is resected during septoplasty, and to confirm possible collapse of the L-strut depending on the dorsal L-strut graft position during augmentation rhinoplasty. This is the first report on the safety of the septal L-strut after dorsal augmentation rhinoplasty. An L-strut model was designed for a numerical analysis. The stress and displacement of the L-strut were evaluated according to the location of an applied load on the dorsal L-strut; the area of contact between the caudal L-strut and the maxillary bone was also considered.

## Materials and Methods

### The Finite Element Method

The FEM is a numerical method that is used to calculate and predict the stress, displacement, and strain energy of a model, whereby the proper element generation (or mesh) for a desired model can be decided and mechanical values can be calculated for each element according to a governing equation. We used the static structure analysis method in Ansys Workbench 2.0 Framework (Taesung ver. 13.0, Korea) as our FEM tool.

### Design of an L-strut model and determination of the mechanical properties

**Fig 1:**
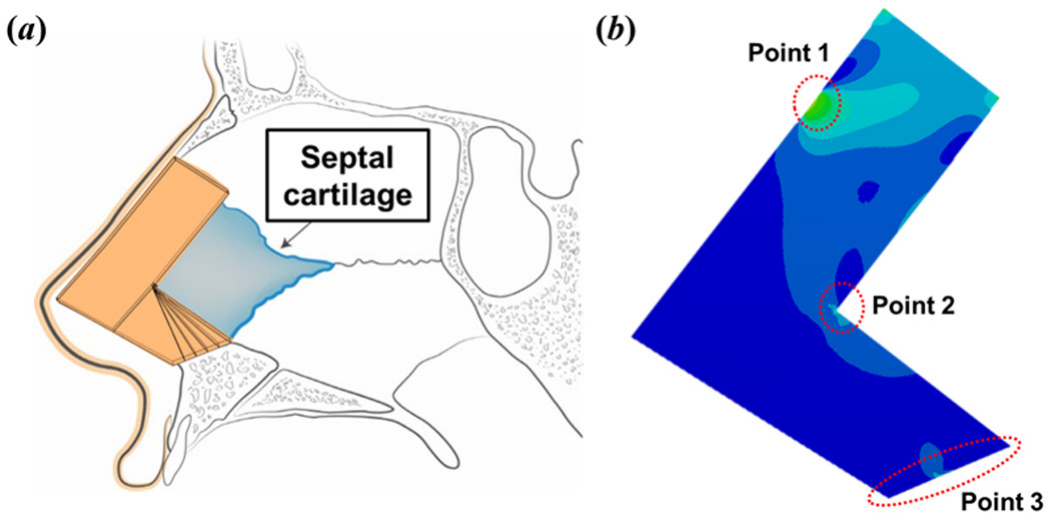
Design of the septal L-strut. **A**. Schematic diagram of the L-strut on the nasal septum in sagittal view. **B**. Point 1 is the location of pressure application. Point 2 is the inner corner of the L-strut. Point 3 is the contact region between the maxillary crest and the caudal septal L-strut.

The septal L-strut was designed as shown in Fig 1A. The width of the L-strut was assumed to be 1 cm, similar to the classical L-strut concept. The regional angle of the septal L-strut was designed based on CT data from 80 patients who underwent trans-sphenoidal skull-base tumor operations at Seoul St. Mary’s Hospital, Seoul, Korea, between July 2008 and September 2011. Approval was obtained from the Institutional Review Board of the Catholic University of Korea, and all patients gave written informed consent. The lengths of the dorsal and caudal L-strut segments were determined from intraoperative measurements of 55 patients in a previous study[21]. In addition, the thickness of the L-strut was determined using cadavers, as described previously[19]. The mechanical properties are described in Table 1; the compressive modulus and Poisson’s ratio were 410 kPa and 0.3, respectively, as the L-strut is septal cartilage[2,22–24].

**Table 1:** Engineering data.

Young’s modulus	4.1 × 10^5^ (Pa)
Poisson’s ratio	0.3

### Boundary condition

**Fig 2:**
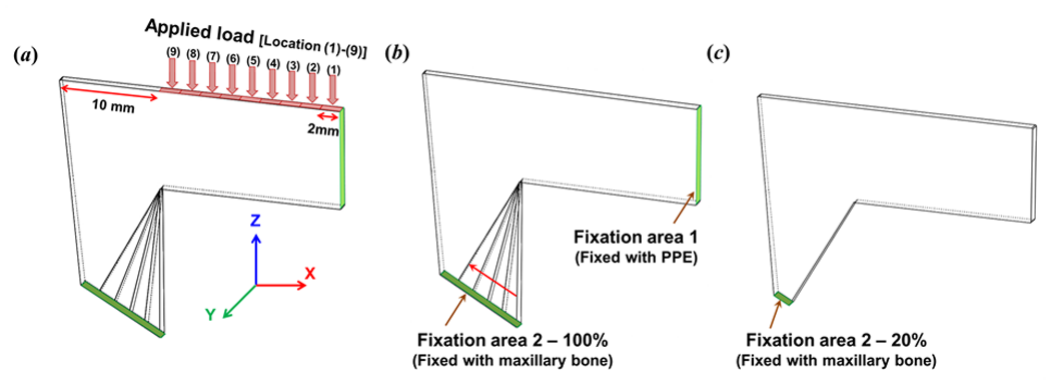
Assumptions for the L-strut condition. **A**. External pressure (0.01N) was applied to the dorsal L-strut in a vertical direction from the rhinion (location 1) to the supratip breakpoint (location 9) at a 2-mm distance. The load was exerted in the 2-mm-wide dorsal segment. The location 9 load condition is the region of the superior point of the L-strut inner corner. **B**. Fixation area 1 is the bonding area with the perpendicular plate of the ethmoid bone (PPE) in the dorsal L-strut. Fixation area 2 is the bonding area with the maxillary crest. The contact percentage in fixation area 2 was assumed to range from 100% to 20%. The 100% condition of fixation area 2 was when the intact 1-cm-wide caudal strut was in contact with the maxillary crest. This simulation was based on partial resection of the caudal septal segment posterior of the anterior nasal spine (ANS) during actual septorhinoplasty. **C**. The 20% condition of fixation area 2 was the 2-mm-wide caudal strut in contact with the maxillary bone posterior to the ANS.

An external load was applied to the L-strut in a vertical direction, as shown in Fig 2A. The load value was 0.01 N, based on the daily load generated in the nose, as it was difficult to exactly measure the daily load as an external force in the nose. The load was applied to a 2-mm-wide dorsal segment of the L-strut at a distance of 2 mm from the rhinion to the supratip breakpoint (i.e., superior point of the L-strut inner corner) under nine pressure location conditions, which were similar to those used in the dorsal augmentation rhinoplasty that is frequently performed in Asian populations (Fig 2A).

The L-strut is attached to the PPE and the maxillary crest, as shown Fig 1. Therefore, we considered this the appropriate test condition, and set up the fixation condition shown in Fig 2B. The fixation condition was the bonding state. We then designed the contact percentage between the L-strut and the maxillary bone, as shown in Fig 2B and 2C.

The L-strut attachment to the maxillary bone is preserved during rhinosurgery, and we termed the contact part as 100% contact (Fig 2B). The 80%, 60%, 55%, 50%, 45%, 40%, and 20% contact percentages were based on 100% contact as the standard. Therefore, the boundary conditions included nine load and eight contact conditions, and we conducted a structural analysis of the L-strut under all boundary conditions to measure the L-strut stress and displacement distributions.

## Results

### Overall L-strut stress distribution

**Fig 3:**
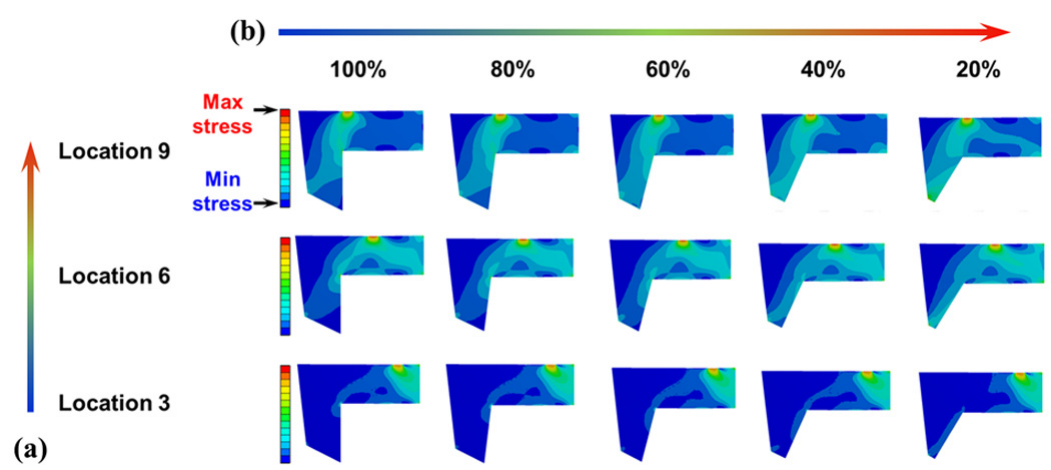
L-strut stress distribution. Red and blue indicate the highest and lowest stresses, respectively. Relatively high stress occurred at point 1 (applied load), point 2 (L-strut inner corner), and point 3 (contact between maxillary bone and the L-strut). Increased stress on the L-strut occurred as the location of the applied load moved caudally (supratip breakpoint) and the contact percentage decreased. **A**. Stress distribution according to the location of the applied load. As the applied load increased near the supratip breakpoint, the stress value increased at point 2 and around point 2. **B**. The stress distribution according to the contact percentage between the L-strut and the maxillary crest. Increased stress occurred in the caudal segment between points 2 and 3 as the contact percentage decreased.

The L-strut model was based on computed tomography (CT) data from 80 patients at our hospital. The same load (0.01 N) was applied to the L-strut. Fig 3 shows the stress distribution in the L-strut model based on the location of the applied load and the percentage contact area between the L-strut and the maxillary crest. The L-strut stress distribution is indicated by several colors. Red and blue indicate the highest and lowest stress levels, respectively. Relatively high stress occurred at point 1 (applied load position), point 2 (L-strut inner corner), and point 3 (contact between maxillary bone and L-strut), as shown in Figs 1B and 3. Green and sky-blue (relatively high stress) appeared in the entire L-strut model, according to the load applied caudally from the keystone area (Fig 3A). In particular, areas of high stress were generated at point 2 (inner corner) and point 3 (contact with maxillary bone) of the L-strut. Therefore, as the load exerted on the nasal dorsum moved from the rhinion to the supratip breakpoint, the distribution of greatest stress on the L-strut changed from the perpendicular plate of the ethmoid bone (PPE) to the inner corner of the L-strut and the inferior part of the caudal strut near the maxillary bone. L-strut stress distribution values increased as the percentage contact area between the L-strut and the maxillary bone decreased, regardless of load condition (Fig 3B). Under these conditions, the stress on the pillar that is connected to the L-strut by the maxillary bone was high. Therefore, the stress at L-strut point 3 increased gradually as the contact area decreased.

### Stress analysis at L-strut points 1–3 according to the location of the applied load

**Fig 4:**
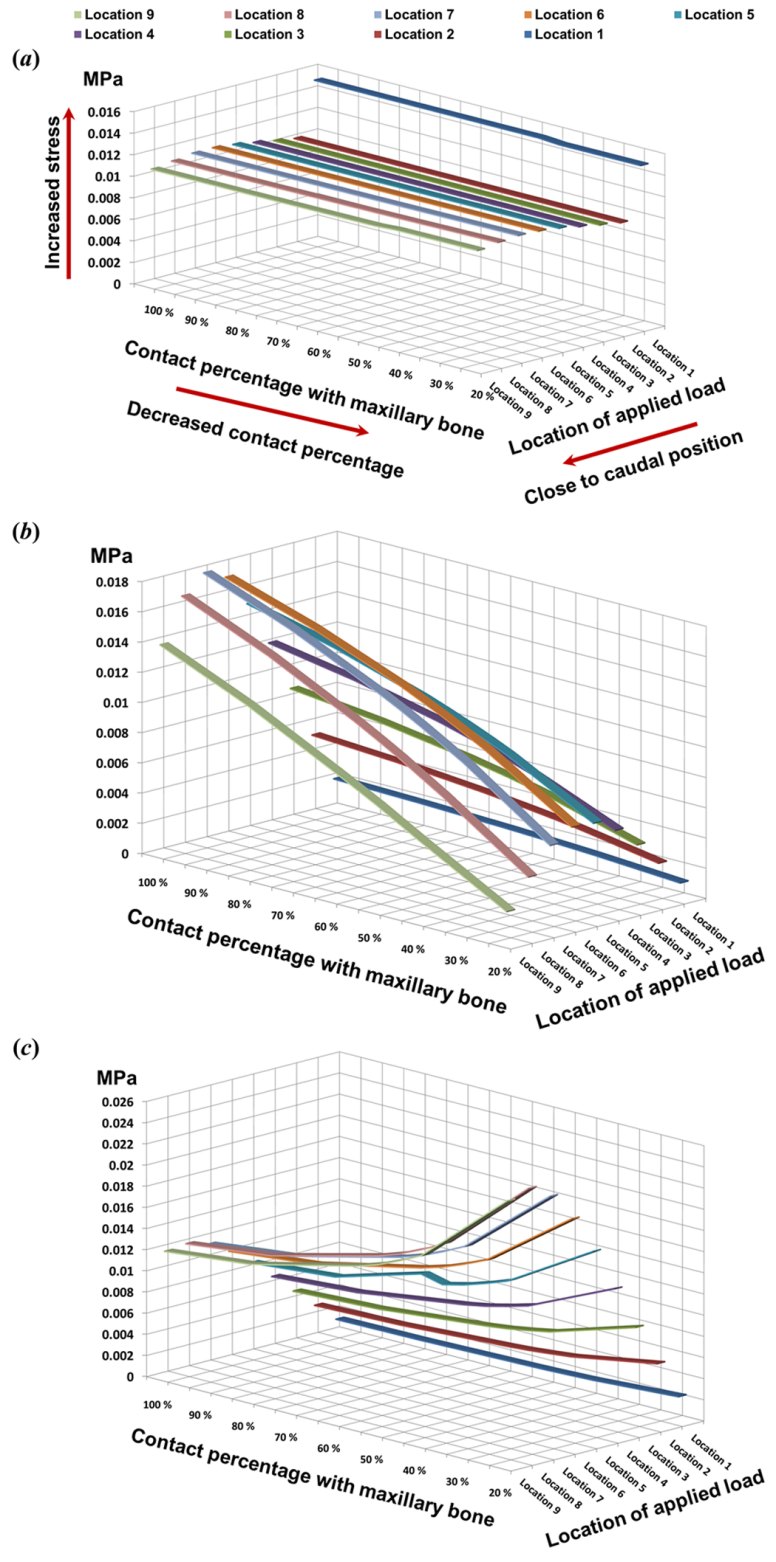
Maximum stress at each point in the L-strut. **A**. The stress was constant at point 1 (pressure exertion region), regardless of the applied load location and the contact percentage with the maxillary crest. **B**. Stress at point 2 decreased gradually after an initial increase, according to changes in the applied load caudally from the rhinion. In particular, the maximum L-strut stress occurred at point 2 under the location 5 and 6 load conditions, regardless of changes in contact percentage. **C**. The stress at point 3 increased when the location of the applied load was changed from the rhinion to the supratip breakpoint.

Fig 4A–4C shows the maximum stress values at L-strut points 1–3, respectively, according to the location of the load and the contact percentage. The stress generated at point 1 remained similar regardless of the load location and the contact percentage, as shown in Fig 4A. This trend is also evident in Fig 3; the stress at point 1 was relatively constant. The stress at point 2 decreased gradually after increasing, according to changes in the load applied caudally from the rhinion (Fig 4B): the maximum stress was under load locations 5 and 6, regardless of changes in the contact percentage. As the location of the applied load moved from location 1 (rhinion) to location 9 (supratip breakpoint), the stress at point 3 increased, as shown in Fig 4C. The stress values increased slowly from location 1 to location 6, and then increased rapidly under the location 7 and 8 load conditions, with similar values under the location 8 and 9 load conditions. The stress distributions for the location 6 to 9 load conditions are shown in Fig 3. The stress values at points 2 and 3 were determined by the location of the applied load and found to be 2- and 2.5-fold higher, respectively, than the applied pressure (0.01 MPa) when the load was subjected to the dorsum of the L-strut from the supratip breakpoint (superior point of the L-strut inner corner) within a 6 mm cephalic length with 20% contact.

### Maximum stress analysis at L-strut points 2 and 3

**Fig 5:**
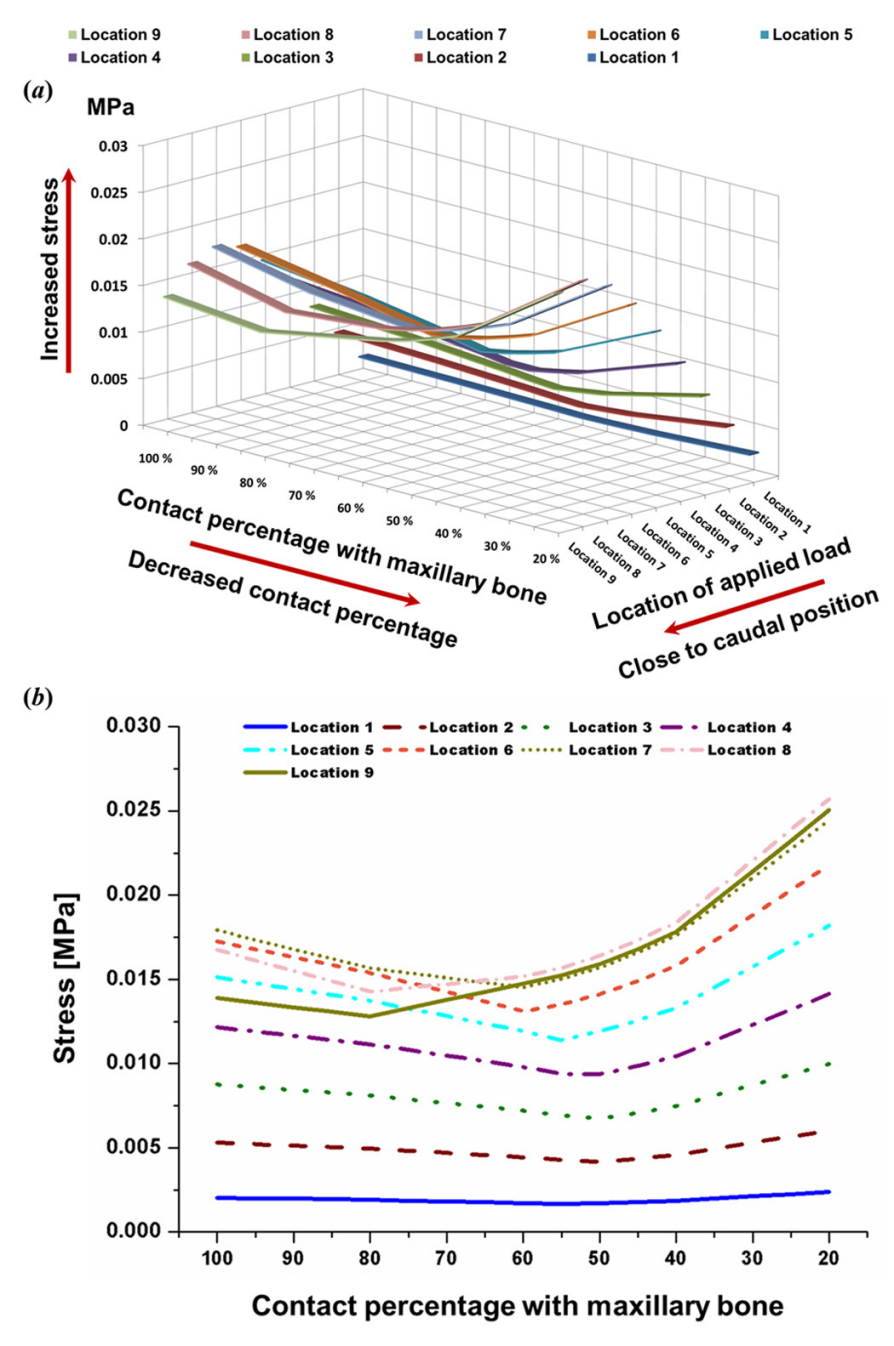
Maximum stress values between stress applied at points 2 and 3 according to the location of the applied load and the contact percentage with the maxillary bone. **A**. Three-dimensional graph. **B**. Two-dimensional graph. Stress values increased gradually as the applied load moved closer to the caudal position. The larger stress values at points 2 and 3 occurred under the location 6–9 load conditions. Stress on the L-strut was dependent on the contact percentage. Stress increased steadily at the later transition point and increased rapidly with contact percentages of 45–20%. Stress values, particularly those under the location 6–9 load conditions, increased rapidly in the 45–20% contact range. Moreover, the maximal L-strut stress values at points 2 and 3 were 2.5-fold larger than that of the applied load.

The effects of the load position and the contact area percentage on stress values at L-strut points 2 and 3, and the maximum stress under each condition, are shown in Fig 5. Stress increased gradually as the applied load neared the caudal position (Fig 5A). The largest stress at points 2 and 3 occurred under the location 6 to 9 load conditions. L-strut stress was dependent on the contact area percentage. Stress values increased steadily at the transition point, while those for contact areas of 45–20% increased rapidly. Stress values, particularly under location 6 to 9 load conditions, steadily increased as the contact percentage decreased from 80–50% and increased rapidly as the contact percentage decreased further to 45–20%. Moreover, the maximum stress values for L-strut points 2 and 3 were 2.5-fold larger than the applied load. Therefore, the L-strut could collapse with contact percentages lower than 45–40%.

### Displacement analysis at L-strut point 2

**Fig 6:**
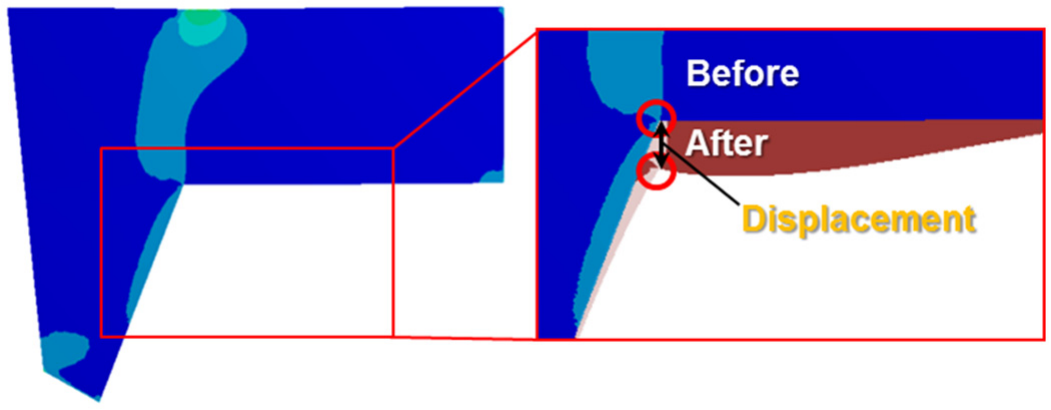
An explanation of displacement at point 2 (L-strut inner corner). The difference in the point 2 location was measured in the Z-axis direction before and after a load was applied to the dorsal L-strut.

**Fig 7:**
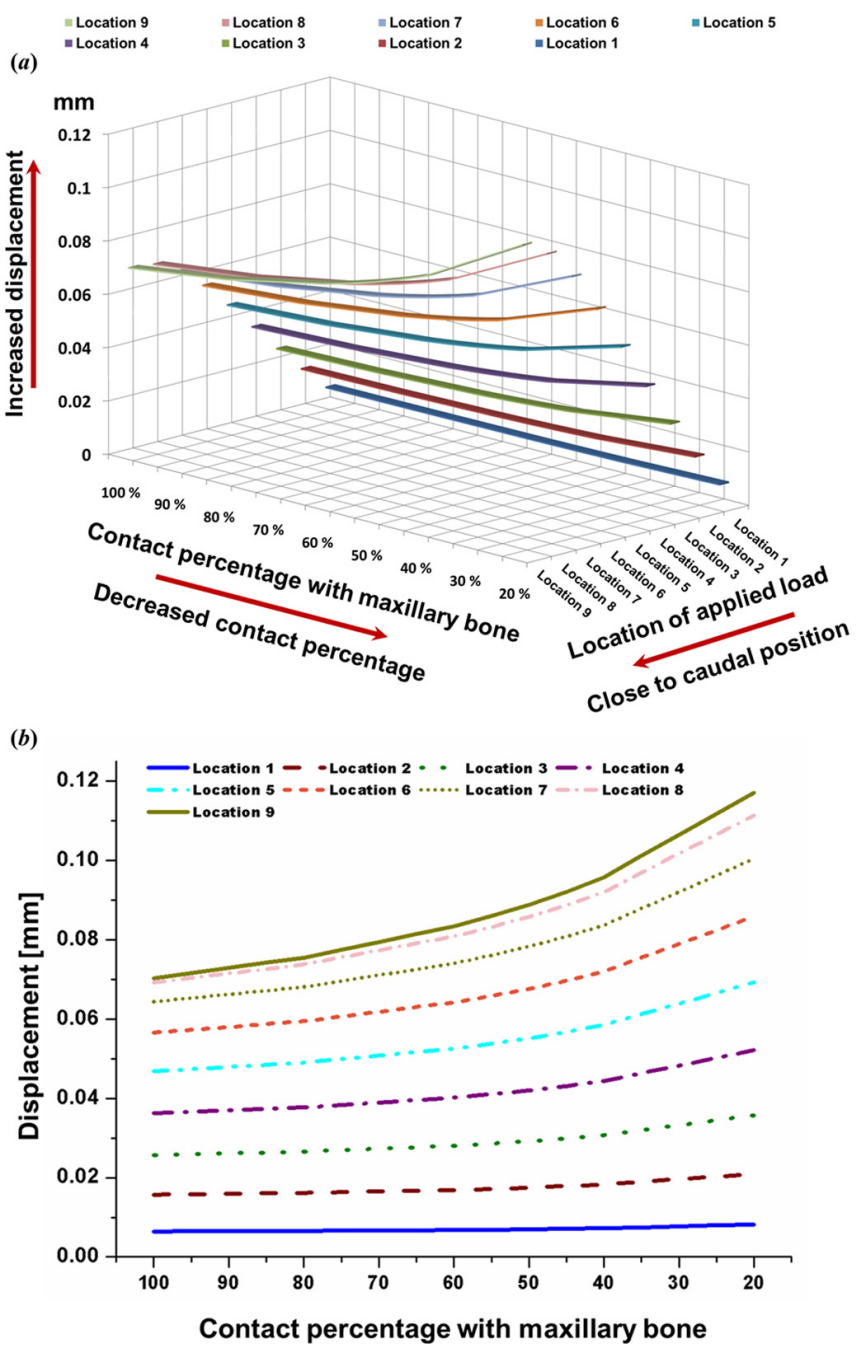
Displacement at point 2. **A**. Displacement values at point 2 rose steadily under the caudal load condition; maximum displacement occurred under the location 8 and 9 load conditions. **B**. Displacement increased as contact percentage decreased, showing rapid displacement in the 45–20% contact range.

Displacement was recorded as the difference at point 2 (L-strut inner corner) between before and after the load was applied at point 1, as shown in Fig 6. Displacement was measured at point 2 in the Z-axis direction (Fig 2A). The displacement values at point 2 rose steadily according to the caudal load condition; maximum displacement occurred under the location 8 and 9 load conditions (Fig 7B). The rate of increase was consistent under the location 1 to 5 load conditions and rose rapidly under the location 6–9 conditions. Moreover, displacement increased as the contact percentage decreased; displacement with 45–20% contact rose rapidly, as shown in Fig 7B. Maximum displacement occurred with 20% contact. Therefore, the L-strut at point 2 with 45–20% areas of contact was deformed and could collapse at contact percentages lower than 45% under the location 6–9 load conditions.

### Stress and displacement analysis under the 100% contact condition

**Fig 8:**
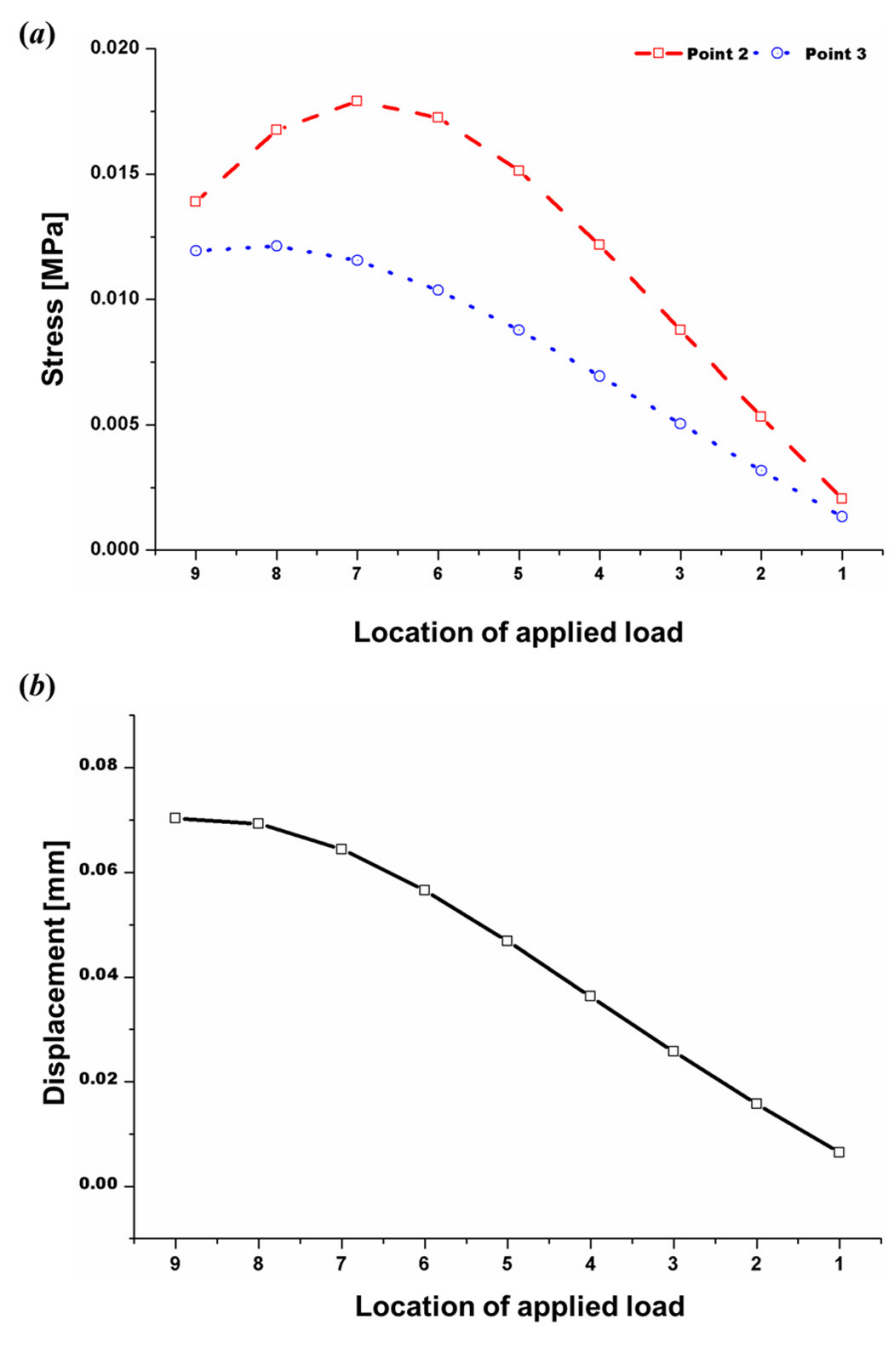
Results for 100% contact between the caudal L-strut and the maxillary crest. **A**. Stress at points 2 and 3 based on the applied load location. Maximum stress occurred at point 3 under the location 8 and 9 loading conditions; stress values at point 2 were higher than those at point 3, with the highest values seen under the location 7 loading condition. **B**. Displacement at point 2 according to the applied load location. As the applied load location changed from location 1 to location 9 (from the rhinion caudally), displacement increased, and maximum values were seen under the location 8 and 9 load conditions (supratip breakpoint).

The 100% contact values were extracted from the results in Fig 7 to simulate an actual dorsal augmentation situation. Fig 8A shows the stress distribution results at L-strut points 2 and 3, according to the location of the applied load, under 100% contact. The stress values at point 3 were maximal under the location 8 and 9 load conditions. Stress values at point 2 were higher than those at point 3, and the highest values at point 2 were seen under the location 7 load condition. The displacement distribution at point 2 was also represented in the 100% contact condition, as shown in Fig 8B. As the applied load moved from location 1 to location 9 (from the rhinion caudally), the displacement values increased, and the maximum values were seen under the location 8 and 9 (supratip breakpoint) load conditions.

## Discussion

We used a FEM simulation to validate the relationship between deformation or possible collapse of the L-strut and the contact percentage/location of an applied load. Complications, such as saddle nose, can occur after septal surgery; however, there has been no report regarding the reasons behind the occurrence of complications, except for anecdotal evidence that relies on individual surgeons’ experience. A 1-cm-wide L-strut is vital for safety and stability during moderate resection of the septal cartilage. Our FEM results indicate that L-strut stress and displacement distributions depending on contact percentage and load conditions can cause severe deformation or collapse of the L-strut.

The stress distribution of the entire L-strut area changed according to the location of the applied load and the percentage contact between the L-strut and the maxillary bone. Greater stresses on the L-strut occurred as the applied load moved toward the caudal position (supratip breakpoint) (Fig 3A) and the contact percentage decreased (Fig 3B). Maximum stress on the L-strut was always generated at points 2 and 3, regardless of the applied load position or the contact percentage. As the applied load neared the supratip breakpoint, the stress value increased around point 2. Larger stress values occurred in the caudal segment between points 2 and 3, depending on the decrease in the contact percentage. Moreover, L-strut stress values depended more on a decrease in the contact percentage than on a change in the applied load position.

We assumed that all mechanical properties of the cartilage were in the elastic region rather than in the plastic region[2,9,10,12,20]. If the mechanical properties of the cartilage in the plastic region had also been considered, the FEM results would portray actual clinical behavior more accurately. However, the possibility of L-strut collapse was predictable based on the FEM results.

In a previous study, we reported that the contact percentage between the L-strut and the maxillary bone is an important factor in collapse of the L-strut; a high possibility of collapse was observed with contact percentages of less than 40%[19]. We concentrated on the effect of contact percentage and subdivided it into 60–40% contact conditions in 5% increments. Larger stress values occurred in the caudal L-strut between points 2 and 3 as the contact percentage decreased from 45 to 20%. Therefore, the possibility of L-strut collapse in the 45–20% contact range was very high, in accordance with the results of our previous study. Compared with the applied stress (0.01 MPa), the stress values at points 2 and 3 were larger, with a rapid increase in the 45–20% contact range, as shown in Fig 4C. The possibility of collapse started to increase at a contact percentage of 45%. Although the position of the transition point changed according to the load location, most transition points among the load conditions were in the 60–40% contact range. The stress values for all load conditions increased at around 50% contact, and stress values increased steeply below 45% contact. Displacement also rose depending on the contact percentage, with the displacement rate increasing with 50–40% contact. Therefore, high deformation energy existed at L-strut point 2, where the stress and displacement values were high. This indicates a high possibility of L-strut collapse in the 45–20% contact range.

Moreover, the degree of change in displacement values at point 2 was relatively small under the location 1–4 load conditions, even with a decrease in the contact percentage. However, displacement values under the location 5–9 load conditions rose according to the contact percentage decrease and increased rapidly with 45–20% contact (Fig 7B). Therefore, the stress (between the L-strut and maxillary bone) and displacement values (at point 2) were 2.5- and 2-fold larger, respectively, than the standard load applied under the 20% contact condition. This result indicates that the contact percentage between the L-strut and maxillary bone affects L-strut collapse. The contact percentage between the caudal L-strut and the maxillary crest should be preserved at > 45% at point 3 to prevent collapse of the L-strut.

The L-strut stress and displacement distributions changed depending on the location of the load on the nasal dorsum and the contact percentage with the maxillary crest. These changes should be considered as a possible factor in L-strut collapse. Deformation of the L-strut increased as the contact percentage between the caudal L-strut and the maxillary crest decreased. With an L-strut width of 1 cm, the ability of the L-strut to maintain its shape decreased when contact with the maxillary bone was 45%, suggesting that the L-strut would inevitably collapse with a contact percentage of less than 45%.

Distortion of the septal cartilage increased when the pressure point changed in the caudal direction of the nasal dorsum. When the applied load was closest to the supratip breakpoint, the stress values at points 2 and 3 increased and the displacement at point 2 increased, as shown in Figs 5B and 7B. The stress values at points 2 and 3 were larger than the applied load under the location 7–9 load conditions (Fig 5B). If the load was applied to the dorsal segment of the L-strut from the supratip breakpoint (superior point of the L-strut inner corner) to within 6 mm cephalically, a 2.5-fold greater stress occurred in the caudal segment of the L-strut around the maxillary bone and the displacement value at point 2 also increased.

Fig 8 shows the L-strut stress and displacement distributions at points 2 and 3 with 100% contact, which preserved the caudal portion of the L-strut. As the results in Fig 8 indicate, if load was applied to the dorsal L-strut in the lower half including the supratip breakpoint the stress and displacement values increased, leading to collapse or transformation of the strut. As these results show, excess augmentation in the lower portions of the nasal dorsum is not desirable; the augmentation material should be distributed widely over the rhinion. In addition, these results indicate that the dorsal augmentation graft must be fixed in an augmentation material pocket on the nasal dorsum to prevent downward displacement of the graft material due to gravity and excess pressure on the supratip breakpoint.

The follow-up period for dorsal augmentation rhinoplasty is generally short, and aesthetic results tend to focus on comparing before and after images. Our results emphasize the importance of long-term maintenance of the nasal dorsal line following a dorsal implant; thus, long-term follow-up is needed after surgery. In a previous study, we found that grafts are occasionally displaced after dorsal augmentation rhinoplasty. These outcomes may be attributed to disruption of the L-strut. Moreover, many studies have reported results using numerous augmentation materials and methods, but relatively few have described septal cartilage design when comparing dorsal augmentation results[5–8]. Accordingly, we suggest that a description of the septal cartilage design should be required when comparing the safety and cosmetic results of dorsal augmentation methods.

The caudal segment of the L-strut must either be preserved perfectly or reinforced with other cartilage during dorsal augmentation. This study is the first step towards provision of patient-customized surgery using FEM. Further scientific development may enable measurements of individual nasal anatomy, which would allow us to predict outcomes in individual patients before surgery. A preoperative FEM simulation may facilitate determination of septal cartilage design and appropriate augmentation material height to provide safe support and good aesthetic results.

FEM software is effective in terms of cost and time because it simulates the mechanical behavior of target tissues and organs. The L-strut model used in this study was prepared with linearly elastic mechanical properties, as we assumed the isotropic properties. We also confirmed L-strut stress and displacement distribution behaviors in one direction and the load in the elastic region. The cartilage of the virtual L-strut had anisotropic, viscoelastic, elastic, and plastic mechanical properties, similar to an actual L-strut. The FEM results would be closer to actual clinical behavior if the mechanical properties of the cartilage, such as the viscoelastic and plastic modulus values, could be identified,. In addition, we conducted the L-strut simulation under a unidirectional load; however, the *in vivo* L-strut is loaded in multiple directions. If a multi-axis load is applied to the L-strut, buckling, deflection, and torsion will occur. Additional studies are needed that include the actual pressure force applied in accordance with the augmentation material (e.g., silicone, Gore-tex, etc.) and the use of realistic septal cartilage configurations. Stress distributions, torsion rates, and moment gradients of the L-strut need to be included in L-strut behavior analyses, using multi-axis loads to simulate actual clinical outcomes.

This study was conducted to analyze the septal L-strut without considering any other factors that could influence strut support. Dorsally and caudally, the cartilaginous septum is interconnected with the upper lateral cartilages and lower lateral cartilages, respectively[4]. Thus, to model more realistically the mechanical behavior of the L-strut using FEM, additional studies incorporating boundary conditions from this surrounding environment in the nasal septum are needed.

## Conclusions

When septoplasty is performed, the inferior portion of the caudal strut must be preserved at > 45% of L-strut width to stabilize the septum. Second, during dorsal augmentation rhinoplasty, because the greatest stress occurs in the inferior caudal L-strut, the strut must either be preserved perfectly or reinforced with other cartilage for safety. Third, the dorsal augmentation material must be fixed in an augmentation pocket to prevent movement of the graft material toward the supratip breakpoint, because downward movement of the graft distorts, disrupts, and may collapse the L-strut. Finally, the FEM is useful for predicting surgical results before surgery and enables patient-customized treatment.

## Supporting Information

S1 Table The regional angle data of the septal L-strut from PNS CT of 80 patients. (XLSX)

## References

[1] Killian G. The submucous window resection of the nasal septum. Ann Otol Rhinol Laryngol. 1905; 14: 363–393.

[2] Lee SJ, Liong K, Lee HP. Deformation of nasal septum during nasal trauma. Laryngoscope. 2010; 120: 1931–1939. 10.1002/lary.21072 20824645

[3] Planas J. The twisted nose. Clin Plast Surg. 1977; 4: 55–67. 856527

[4] Kim DW, Gurney T. Management of naso-septal L-strut deformities. Facial Plast Surg. 2006; 22: 9–27. 16732500 10.1055/s-2006-939948

[5] Jin HR, Won TB. Recent advances in Asian rhinoplasty. Auris Nasus Larynx. 2011; 38: 157–164. 10.1016/j.anl.2010.05.014 20728295

[6] Toriumi DM, Swartout B. Asian rhinoplasty. Facial Plast Surg Clin N Am. 2007; 15: 293–307. 10.1016/j.fsc.2007.04.003 17658425

[7] Jang YJ, Alfanta EM. Rhinoplasty in the Asian nose. Facial Plast Surg Clin N Am. 2014; 22: 357–377. 10.1016/j.fsc.2014.04.001 25049122

[8] Toriumi DM, Pero CD. Asian rhinoplasty. Clin Plast Surg. 2007; 37: 335–352. 10.1016/j.cps.2009.12.008 20206750

[9] Mau T, Mau ST, Kim DW. Cadaveric and engineering analysis of the septal L-strut. Laryngoscope. 2007; 117: 1902–1906. 17721403 10.1097/MLG.0b013e3181255ec4

[10] Lee SJ, Liong K, Tse KM, Lee HP. Biomechanics of the deformity of septal L-Struts. Laryngoscope. 2010; 120: 1508–1515. 10.1002/lary.20976 20564665

[11] Koike T, Wada H, Kobayashi T. Modeling of the human middle ear using the finite-element method. J Acoust Soc Am. 2002; 111: 1306–1317. 11931308 10.1121/1.1451073

[12] Jung JW, Park JH, Hong JM, Kang HW, Cho DW. Octahedron pore architecture to enhance flexibility of nasal implant-shaped scaffold for rhinoplasty. Int J Precis Eng Manuf. 2014; 15: 2611–2616.

[13] Gan RZ, Feng B, Sun Q. Three-dimensional finite element modeling of human ear for sound transmission. Ann Biomed Eng. 2004; 32: 847–859. 15255215 10.1023/b:abme.0000030260.22737.53

[14] Vampola T, Laukkanen AM, Horacek J, Svec JG. Vocal tract changes caused by phonation into a tube: a case study using computer tomography and finite-element modeling. J Acoust Soc Am. 2011; 129: 310–315. 10.1121/1.3506347 21303012

[15] Manuel CT, Leary RP, Protsenko DE, Wong BJ. Nasal tip support: a finite element analysis of the role of the caudal septum during tip depression. Laryngoscope. 2014; 3: 649–665. 10.1002/lary.24321 PMC4364034 23878007

[16] Shamouelian D, Leary RP, Manuel CT, Harb R, Protsenko DE, Wong BJ. Rethinking nasal tip support: A finite element analysis. Laryngoscope. 2015; 125: 326–330. 10.1002/lary.24845 25130506 PMC4304991

[17] Jung JW, Yi HG, Kang TY, Yong WJ, Jin SW, Yun WS, et al Evaluation of the effective diffusivity of a freeform fabricated scaffold using computational simulation. J Biomech Eng-Trans. ASME. 2013; 135: 084501.

[18] Kundu J, Shim JH, Jang J, Kim SW, Cho DW. An additive manufacturing-based PCL—alginate—chondrocyte bioprinted scaffold for cartilage tissue engineering. J Tissue Eng Regen Med. 2013 Online published. 10.1002/term.1682 23349081

[19] Lee JS, Lee DC, Ha DH, Kim SW, Cho DW. Redefining the septal L-strut in septal surgery. PLoS One 2015; 10, e0119996 10.1371/journal.pone.0119996 25803842 PMC4372341

[20] Liong K, Lee SJ, Lee HP. Preliminary deformational studies on a finite element model of the nasal septum reveals key areas for septal realignment and reconstruction. J Med Eng. 2013; 250274 (8 pp). 10.1155/2013/250274 27006910 PMC4782633

[21] Kim JS, Khan NA, Song HM, Jang YJ. Intraoperative measurements of harvestable septal cartilage in rhinoplasty. Ann Plast Surg. 2010; 65: 519–523. 10.1097/SAP.0b013e3181d59f95 20948417

[22] Glasgold MJ, Kato YP, Christiansen D, Hauge JA, Glasgold AI, Silver FH. Mechanical properties of septal cartilage homografts, Otolaryngol. Head Neck Surg. 1988; 99: 374–379. 10.1177/019459988809900404 3148886

[23] Richmon JD, Sage AB, Wong VW, Chen AC, Pan C, Sah RL, et al Tensile biomechanical properties of human nasal septal cartilage. Am J Rhinol. 2005; 19: 617–622. 16402652

[24] Richmon JD, Sage AB, Wong VW, Chen AC, Sah RL, Watson D. Compressive biomechanical properties of human nasal septal cartilage. Am J Rhinol. 2006; 20: 496–501. 17063745 10.2500/ajr.2006.20.2932

